# Subtractionless compressed-sensing-accelerated whole-body MR angiography using two-point Dixon fat suppression with single-pass half-reduced contrast dose: feasibility study and initial experience

**DOI:** 10.1186/s12968-023-00953-w

**Published:** 2023-07-20

**Authors:** Qing Fu, Zi-qiao Lei, Jing-yang Li, Jia-wei Wu, Xiao-ming Liu, Wen-liang Fan, Peng Sun, Jia-zheng Wang, Ding-xi Liu, Fan Yang, Chuan-sheng Zheng, Xiang-chuang Kong

**Affiliations:** 1 Department of Radiology, Union Hospital, Tongji Medical College, Huazhong University of Science and Technology, Jiefang Avenue #1277, Wuhan, 430022 Hubei Province China; 2 Hubei Province Key Laboratory of Molecular Imaging, Wuhan, 430022 China; 3 Philips Healthcare, Beijing, 100600 China

**Keywords:** Whole-body magnetic resonance angiography (WBMRA), Magnetic resonance imaging (MRI), Compressed sensing, Multi-echo Dixon (mDixon)

## Abstract

**Purpose:**

To investigate the feasibility and clinical utility of a compressed-sensing-accelerated subtractionless whole-body MRA (CS-WBMRA) protocol with only contrast injection for suspected arterial diseases, by comparison to conventional dual-pass subtraction-based whole-body MRA (conventional-WBMRA) and available computed tomography angiography (CTA).

**Materials and methods:**

This prospective study assessed 86 patients (mean age, 56 years ± 16.4 [standard deviation]; 25 women) with suspected arterial diseases from May 2021 to December 2022, who underwent CS-WBMRA (n = 48, mean age, 55.9 years ± 16.4 [standard deviation]; 25 women) and conventional-WBMRA (n = 38, mean age, 48 years ± 17.4 [standard deviation]; 20 women) on a 3.0 T MRI after random group assignment based on the chronological order of enrolment. Of all enrolled patients administered the CS-WBMRA protocol, 35% (17/48) underwent CTA as required by clinical demands. Two experienced radiologists independently scored the qualitative image quality and venous enhancement contamination. Quantitative image assessment was carried out by determining and comparing the apparent signal-to-noise ratios (SNRs) and contrast-to-noise ratios (CNRs) of four representative arterial segments. The total examination time and contrast-dose were also recorded. The independent samples t-test or the Wilcoxon rank sum test was used for statistical analysis.

**Results:**

The overall scores of CS-WBMRA outperformed those of conventional-WMBRA (3.40 ± 0.60 vs 3.22 ± 0.55, P < 0.001). In total, 1776 and 1406 arterial segments in the CS-WBMRA and conventional-WBMRA group were evaluated. Qualitative image scores for 7 (of 15) vessel segments in the CS-WMBRA group had statistically significantly increased values compared to those of the conventional-WBMRA groups (P < 0.05). Scores from the other 8 segments showed similar image quality (P > 0.05) between the two protocols. In the quantitative analysis, overall apparent SNRs were significantly higher in the conventional-WBMRA group than in the CS-WBMRA group (214.98 ± 136.05 vs 164.90 ± 118.05; P < 0.001), while overall apparent CNRs were not significantly different in these two groups (CS vs conventional: 107.13 ± 72.323 vs 161.24 ± 118.64; P > 0.05). In the CS-WBMRA group, 7 of 1776 (0.4%) vessel segments were contaminated severely by venous enhancement, while in the convention-WBMRA group, 317 of 1406 (23%) were rated as severe contamination. In the CS-WBMRA group, total examination and reconstruction times were only 7 min and 10 min, respectively, vs 20 min and < 30 s for the conventional WBMRA group, respectively. The contrast agent dose used in the CS-WBMRA protocol was reduced by half compared to conventional-WBMRA protocol (18.7 ± 3.5 ml vs 37.2 ± 5.4 ml, P = 0.008).

**Conclusion:**

The CS-WBMRA protocol provides excellent image quality and sufficient diagnostic accuracy for whole-body arterial disease, with relatively faster workflow and half-dose reduction of contrast agent, which has greater potential in clinical practice compared with conventional-WBMRA.

## Background

Contrast-enhanced magnetic resonance angiography (CE-MRA) is widely applied for detecting peripheral arterial and venous diseases with high accuracy [1–3]. Takayasu’s arteritis, arterio-sclerotic cardiovascular diseases (ASCVD), polyarteritis nodosa (PAN) and peripheral vascular disease (PVD) are systemic vasculitides that predominantly affect medium-sized muscular arteries and often involve small muscular arteries in multiple anatomical regions [4]. However, to achieve a whole-body scan within the arterial phase, current techniques typically require two sets of scans and contrast injections (i.e., the dual-pass method), due to the short contrast flow time (< 5 s) over the abdominal aorta [5]. Otherwise, after the first-pass, visualization of the thoracic and upper extremity vasculature is contaminated by residual contrast agent from the first injection [6–8]. Furthermore, incorrect subtraction caused by the patient’s movement seriously interferes with the visualization of blood vessels and clinical diagnosis. Moreover, prolonged imaging time and complex operations tend to degrade image quality if patients cannot tolerate the long examination time, especially for symptomatic elderly cases, or in case of inadequate experience of the MR technician.

On the other hand, the Dixon-based method has been successfully applied in peripheral MRA for depicting PVD without the need for image subtraction at 1.5 T [9] and 3.0 T, which provides homogenous fat-suppression even in a large FOV due to its insensitivity to B0 and B1 heterogeneities [10]. Multi-echo Dixon (mDixon) [11] is a novel water-fat separation strategy with multiple gradient-echoes and a more flexible echo time (TE) design, which allows better signal-to-noise ratio (SNR), higher spatial resolution, shorter scan time, and better vessel-to-background contrast compared with the common subtraction method [9, 12]. Based on the above studies, we hypothesized the feasibility of mDixon for whole-body CE-MRA.

Parallel imaging (PI), e.g., sensitivity encoding (SENSE), is usually employed to accelerate data acquisition by under-sampling k-space data [13–15], and its application for whole-body CE-MRA in combination with a single contrast injection proved to be feasible within a short scan time [15]. However, the PI acceleration factor is typically limited to below threefold to avoid image quality degradation [16–18]. In the past decade, compressed sensing (CS) has attracted increasing attraction for considerably reducing scan time while maintaining MR image quality [19–22]. It accelerates MRI acquisition, exploiting image sparsity via a non-uniform under-sampling pattern and nonlinear reconstruction [20–23]. MRA data are inherently sparse and therefore suitable for the CS technology [23, 24], with proven applications in time-of-flight MRA (TOF-MRA) and CE-MRA [23, 25, 26]. However, few studies have assessed the performance of CS for whole-body CE-MRA in a clinical population.

Here we hypothesize that a subtractionless single-pass (0.15 mmol/kg dose) whole-body CE-MRA protocol is clinically feasible, with the CS-accelerated mDixon MRA sequence. Therefore, this study aimed to evaluate the feasibility of the developed subtractionless single-pass CS-WBMRA protocol, and to compare this approach with the conventional dual-pass subtraction-based WBMRA protocol and available computed tomography angiography (CTA) for clinical performance.

## Materials and methods

### Patient inclusion

This study followed the Declaration of Helsinki and was approved by the Ethics Committee of Union Hospital, Tongji Medical College, Huazhong University of Science and Technology (No.2021-0122). Participants were informed of the MR procedures and written informed consent was obtained before any contrast-enhanced MRA examination.

From May 2021 to December 2022, totally 86 patients with suspected arterial diseases were enrolled and assigned to 2 groups randomly based on the chronological order of enrolment: 38 (44%) control subjects (18 males and 20 females, 47.7 ± 17.4 years old) were assigned to undergo conventional-WBMRA; 48 (56%) patients (23 males and 25 females, 55.9 ± 16.4 years old) assigned to undergo CS-WBMRA protocol, of whom 17 (35%) patients (9 males and 8 females, averaging 61.4 years old; 11 patients with arteriosclerosis and 6 with systemic vasculitides) also underwent CTA based on clinical demands. Exclusion criteria were contraindications for contrast-enhanced MRI (known prior adverse reactions to the contrast agent, claustrophobia, pregnancy in women, severe dyspnea, continuous cough, inability to establish intravenous access, or acute or chronic severe renal impairment with eGFR < 30 ml/min/1.73 m^2^).

### MRI protocols

All MRA examinations were performed on a 3.0 T MR scanner (Ingenia CX, Philips Healthcare, Best, the Netherlands) with a body coil for radiofrequency transmission and a 20-channel head-neck coil plus two 32-channel abdomen coils for signal reception, which provided coverage from head vertex to ankle level. Patients were placed in the supine position with headfirst into the bore of the magnet. The patient’s arms were kept close to bilateral sides, and the ankles were elevated with a folded soft sponge. The lateral peripheral edges of surface coils were fixed to the MRI table with belts. During the whole procedure of MR imaging, patients were required to stay still and to cooperate in breathing (during thorax and abdomen imaging) to avoid motion artifacts.

**Fig. 1:**
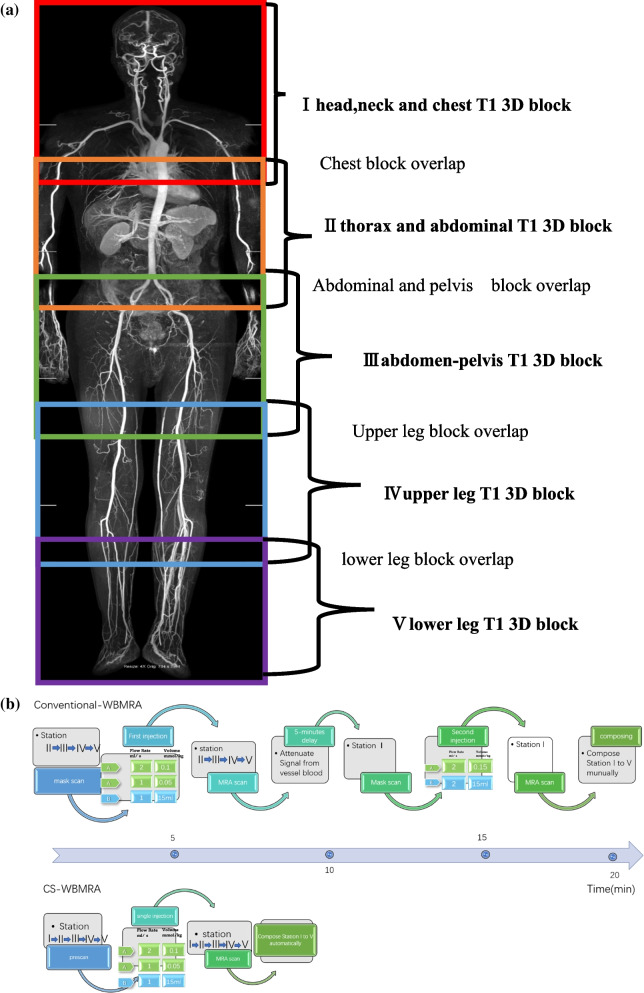
**A** positioning FOV for WBMRA planning. Each block is the same FOV (450 mm × 450 mm) and overlap between every block for reducing geometry distortion. **B** Flow chart of the scan and contrast injection strategies of the two MRA protocols in the timeline

Conventional-WBMRA images were acquired with the 3D T1-FFE sequence in a successive order based on the acquisitions of 4 or 5 overlapped stations with head-to-ankle coverage (Fig. 1A): station I (head-thorax), station II (thorax-abdomen), station III (abdomen-pelvis), station IV (upper leg) and station V (lower leg). The field-of-view (FOV) of each station was 450 mm with 90–130 mm overlap depending on patient height to avoid a regions with low signals between two consecutive stations. Patients below 160 cm were scanned with 4-stations, while 5 stations were used for the remaining cases. Gadopentetate dimeglumine (Magnevist; Bayer Schering Pharma AG) was injected into the right antecubital vein via a 22‑gauge needle with a power injector (Spectris; MedRad). For both MRA protocols, image acquisition was initiated with the contrast agent arriving in pulmonary arteries by a real-time contrast bolus tracking technique (Bolus Track; Philips Healthcare, Best, The Netherlands). For the whole-body MRA scanning procedure, localizer images were obtained in all stations to cover the whole body from head vertex to ankles. Then, a phase-contrast vessel scout for each station was scanned for localization, followed by contrast-enhanced MRA sequences. The time for table movement between adjacent stations was about 1 s. The mean time for the entire examination from patient positioning to examination end and the time required for image reconstruction of both MRA methods were recorded respectively.

Detailed scan parameters for CS-WBMRA are listed in Table 1. Only one single-pass contrast injection was applied in CS-WBMRA, and the detailed injection strategy is depicted in the flow chart (Fig. 1B): a single dose (0.1 mmol/kg) contrast agent was injected at a flow rate of 2.0 ml/s, followed by a half dose (0.05 mmol/kg) at 1.0 ml/s, with a final saline bolus of 15 ml at 1.0 ml/s. After arterial acquisition from head to ankle, the venous phase was immediately scanned successively from calf to head with the same sequences described above.

**Table 1:** Detailed parameters of the two-point Dixon sequence in the CS-WBMRA protocol

Parameter	Station I	Station II	Station III	Station IV	Station V
Repetition time (TR, ms)	4.0	4.2	3.8	4.2	4.5
First/second echo time (TE1/TE2, ms)	1.38/2.50	1.48/2.60	1.32/2.40	1.46/2.70	1.55/2.90
Flip angle (FA, °)	20	20	20	20	20
Field of view (FOV, mm^2^)	450 × 450	450 × 450	450 × 450	450 × 450	450 × 450
Voxel size (mm^3^)	1.1 × 1.2 × 1.2	1.2 × 1.2 × 1.5	1.2 × 1.2 × 1.5	1.0 × 1.0 × 1.0	0.9 × 0.9 × 1.0
Slice number	130	125	125	130	110
Compressed sensing factor	10	10	10	10	8
Bandwidth (Hz)	1225.5	1329.8	1329.8	1106.2	1000
k-space filling mode	Reverse centric	Centric	Centric	Centric	Centric
Half scan	0.8	0.8	0.8	0.8	0.8
Need for breath-hold	Yes	Yes	No	No	No
Acquisition time (s)	9.8	11.7	10.8	12.5	15.6

CS-WBMRA, compressed sensing whole body MR angiography

In conventional-WBMRA protocol [27], pre- and post-contrast images of all regions were acquired using the fast field echo 3D sequence (FFE), and detailed parameters are listed in Table 2. Subtraction of post-contrast and pre-contrast images was performed for conventional WBMRA imaging. In this protocol, the contrast agent was injected by a biphasic injection strategy as illustrated in Fig. 1B. The first injection was used to cover thorax-abdomen-pelvis-upper leg-lower leg with 0.1 mmol/kg contrast agent injected at a flow rate of 2.0 ml/s; then, 0.05 mmol/kg contrast agent was injected at a flow rate of 1.0 ml/s, followed by a final 15 ml saline bolus at a rate of 1.0 ml/s. After arterial acquisition from thorax to lower leg (station II-V), the venous phase was immediately scanned successively in the opposite direction with the sequences described above. Arterial and venous subtracted MRA images were reconstructed automatically. Five minutes later, a second injection was used for imaging the head-neck area (station I), 0.15 mmol/kg contrast agent was injected at a rate of 2.5 ml/s, followed by a 15 ml saline bolus at a rate of 2.5 ml/s. The venous phase was scanned immediately following the arterial phase. In total, 0.3 mmol/kg contrast agent was applied in this protocol.

**Table 2:** Detailed parameters of the turbo fast low-angle shot 3D sequence used in the conventional-WBMRA protocol

Parameter	Station I	Station II	Station III	Station IV	Station V
Repetition time (TR, ms)	3.9	3.9	3.9	3.9	3.9
Echo time (TE, ms)	1.29	1.26	1.26	1.34	1.40
Flip angle (FA, °)	20	20	20	20	20
Field of view (FOV, mm^2^)	450 × 450	450 × 450	450 × 450	450 × 450	450 × 450
Voxel size (mm^3^)	1.1 × 1.2 × 1.2	1.2 × 1.2 × 1.5	1.2 × 1.2 × 1.5	1.0 × 1.0 × 1.0	0.9 × 0.9 × 1.0
Slice number	125	125	125	130	110
Sense acceleration factor	4.3	4	4	4.5	4.5
Bandwidth (Hz)	1225.5	1329.8	1329.8	1106.2	1000
k-space filling mode	Centra	Centra	Centra	Centra	Centra
Phase partial Fourier	0.75	0.75	0.75	0.75	0.75
Need for breath-hold	Yes	Yes	No	No	No
Acquisition time (s)	18.2	20	20	20	18.7

*WBMRA* whole body MR angiography

### Computed tomography angiography protocol

For each of the 17 patients submitted to CTA, one of the four protocols (intracranial, carotid, aortic, or lower extremity) was performed according to clinical diagnostic purpose, with a 128-multislice CT system (MSCT) (SOMATOM AS +; Siemens Healthcare, Germany). A bolus of 45–100 ml non-ionic contrast agent (Iodixanol 320 mg I/ml, HENGRUI MEDICINE, China) was injected with a power injector (Stellant, CT injection system MedRad, Warrendale, Pennsylvania, USA) via a 20-gauge intravenous antecubital catheter at 3–4.5 ml/s, followed by a 40–50 ml saline bolus at 3–4.5 ml/s. The triggering threshold was 80–300 Hounsfield Unit (HU) and a region of interest (ROI) was placed within the internal carotid artery, the ascending aorta, or the descending aorta, depending on the CTA protocol. Automatic CTA scanning was triggered 2–6 s after the ROI attenuation value exceeded the above thresholds. Scanning parameters were: tube voltage, 100–120 kV; automatic tube current modulation (CARE Dose 4D); pitch, 0.18–8; collimation, 0.6 mm × 128; reconstruction thickness, 0.75–3 mm (0.6–3 mm increments); gantry rotation time, 0.25 s.

### Evaluation

For image analysis, the arterial tree was divided into 37 vessel segments [6]. For qualitative image quality evaluation, the arterial tree was divided into 22 segments, including bilateral common carotid arteries, bilateral vertebral arteries, brachiocephalic trunk, thoracic aorta, abdominal aorta, bilateral renal arteries, celiac artery, superior mesenteric artery, bilateral common iliac arteries, bilateral external iliac arteries, bilateral femoral arteries, bilateral popliteal arteries, bilateral anterior tibial arteries, bilateral posterior tibial arteries and bilateral peroneal arteries.

For each subject, merged maximum intensity projections (MIP) of all stations were generated (MobiView, R5.71 version, Philips Healthcare, Best, the Netherlands) to provide an overview of the entire arterial vasculature. Two experienced radiologists (8 and 16 years of experience) analyzed the CE-MRA images acquired with both MRA protocols. Both MIP images in different projection angles and source images were evaluated. The radiologists were blinded to patient information and available CTA data. In case of disagreement, a third experienced radiologist was involved to make a final decision.

### Qualitative image quality analysis

The overall image quality of both MRA datasets and the visualization of each arterial segment were evaluated by the two above radiologists in consensus using a 4-point scoring scale (Table 3) [7, 9], with a score ≥ 3 reflecting an accepted diagnostic level. Venous enhancement contamination was assessed with a 3-point scale (Table 3), with a score of 1 or 2 considered to be useful for diagnosis.

**Table 3:** Criteria for qualitative image quality comparisons between the two MRA protocols

Overall image quality and visualization of each arterial segment
1, Poor image quality, nondiagnostic grade, no arteries visible 2, Fair image quality, not all arterial segments evaluable due to image blurring/artifacts or inadequate arterial enhancement for confident diagnosis 3, Acceptable image quality with minimal blurring/artifacts and adequate arterial enhancement, all arterial segments evaluable for definite diagnosis 4, Good to excellent image quality, sharply defined vessel borders and arterial enhancement, all arterial segments evaluable for highly confident diagnosis without artefacts
Contaminating venous enhancement
1, None or minimal venous signals 2, Mild-to-moderate, not interfering with the definite diagnosis 3, Severe, interfering with diagnosis

### Quantitative image analysis

The image quality of both MRA protocols was analyzed objectively by calculating the apparent signal-to-noise ratio (SNRs) and contrast-to-noise ratio (CNRs) of four representative arterial segments (common carotid arteries, abdominal aorta, common femoral arteries and popliteal arteries). Apparent SNRs and CNRs were determined as follows: SNR = SI_vessel_/SD and CNR = (SI_vessel_ − SI_background_)/SD; SI_vessel_, arterial signal intensity, was measured using a user-defined region-of-interest (ROI) in the center of the vessel, SI_background_ was the background signal intensity with identical ROI size of the region adjacent to the vessel, and SD is the standard deviation of air outside the patient. Signals for artifacts, vessel borders and atherosclerotic plaques were avoided for all measurements. To avoid bias, each measurement was carried out 2–3 times, and averaged values were used for further evaluation.

### Diagnostic accuracy

For each subject, a subset of CS-WBMRA images were reviewed against the available CTA examinations by the above two radiologists based on segment-to-segment analysis for detecting arterial pathologies as follows [1, 8]: 1, normal; 2, minimal to mild stenosis with luminal narrowing below 50%; 3, severe stenosis with luminal narrowing 50–100%; 4, arterial occlusion or aneurysmal disease. The overall sensitivity and specificity of CS-WBMRA in the detection of significant stenosis (luminal narrowing > 50%) were obtained with CTA as the reference standard.

### Statistical analysis

Statistical analysis was performed with SPSS (IBM SPSS 22, IBM Corp., Armonk, New York, USA). Continuous variables are mean ± standard deviation (SD). If the apparent SNRs and CNRs for both MRA groups conformed to normal distribution (Shapiro–Wilk test), the independent samples t-test was used; otherwise, the Wilcoxon rank sum test was used. P < 0.05 was considered statistically significant.

## Results

**Fig. 2:**
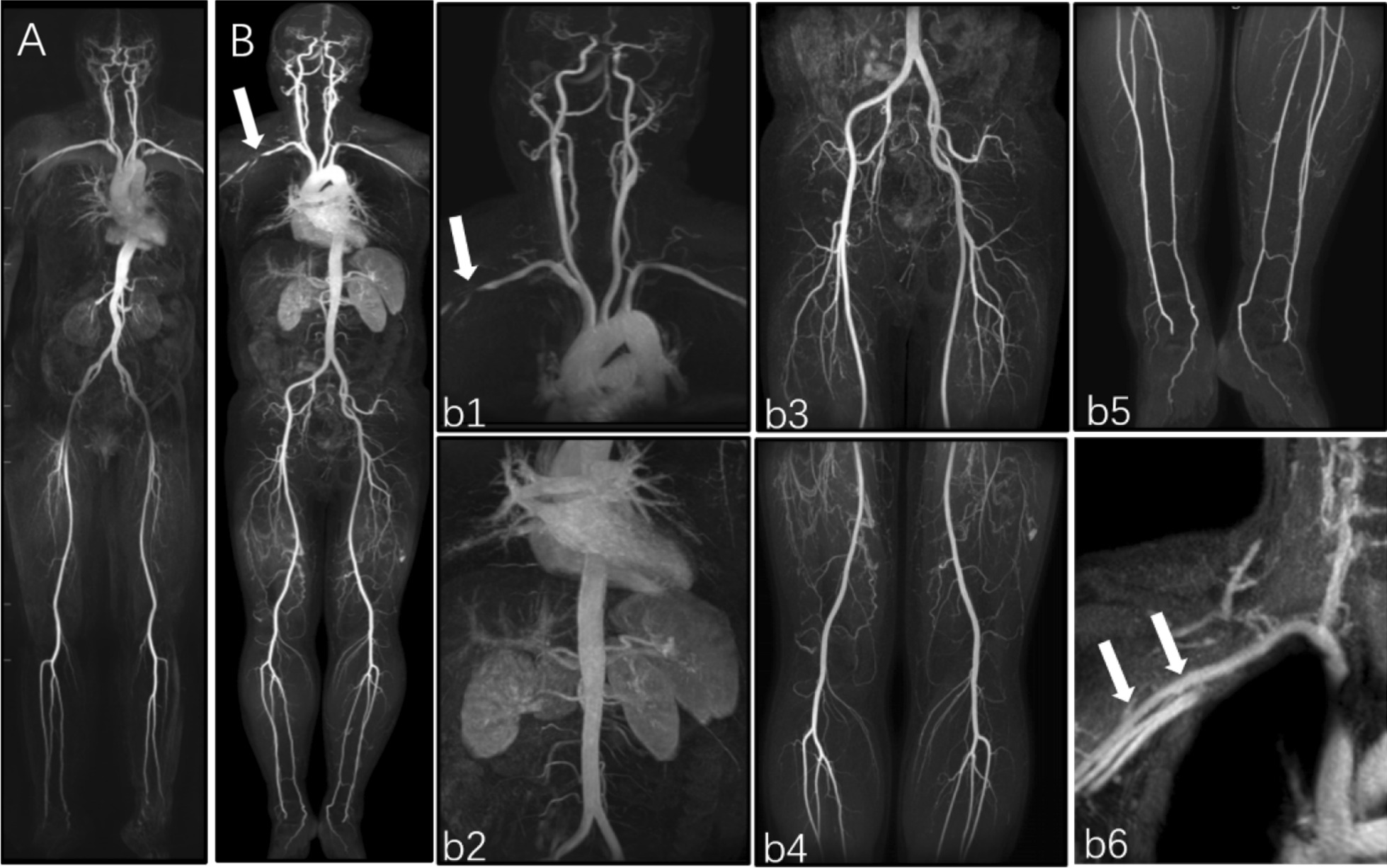
Whole-body MRA MIP (**A**) in a female healthy volunteer (47 years old) assessed by conventional MRA and Whole-body CS-WBMRA MIP (**B**) in a male patient (50 years old) examined by subtractionless CS-WBMRA. Both showed excellent image qualities in depicting whole-body arterial vessels. Successive images of five stations in CS-WBMRA, including station I (b1), station II (b2), station III (b3), station IV (b4) and station V (b5), display arteries from head-neck to ankle very clearly. Interestingly, there seemed to be an occlusion in the junction between the right axillary artery, but it was identified as normal in the venous phase of CS-WBMRA (b6)

**Fig. 3:**
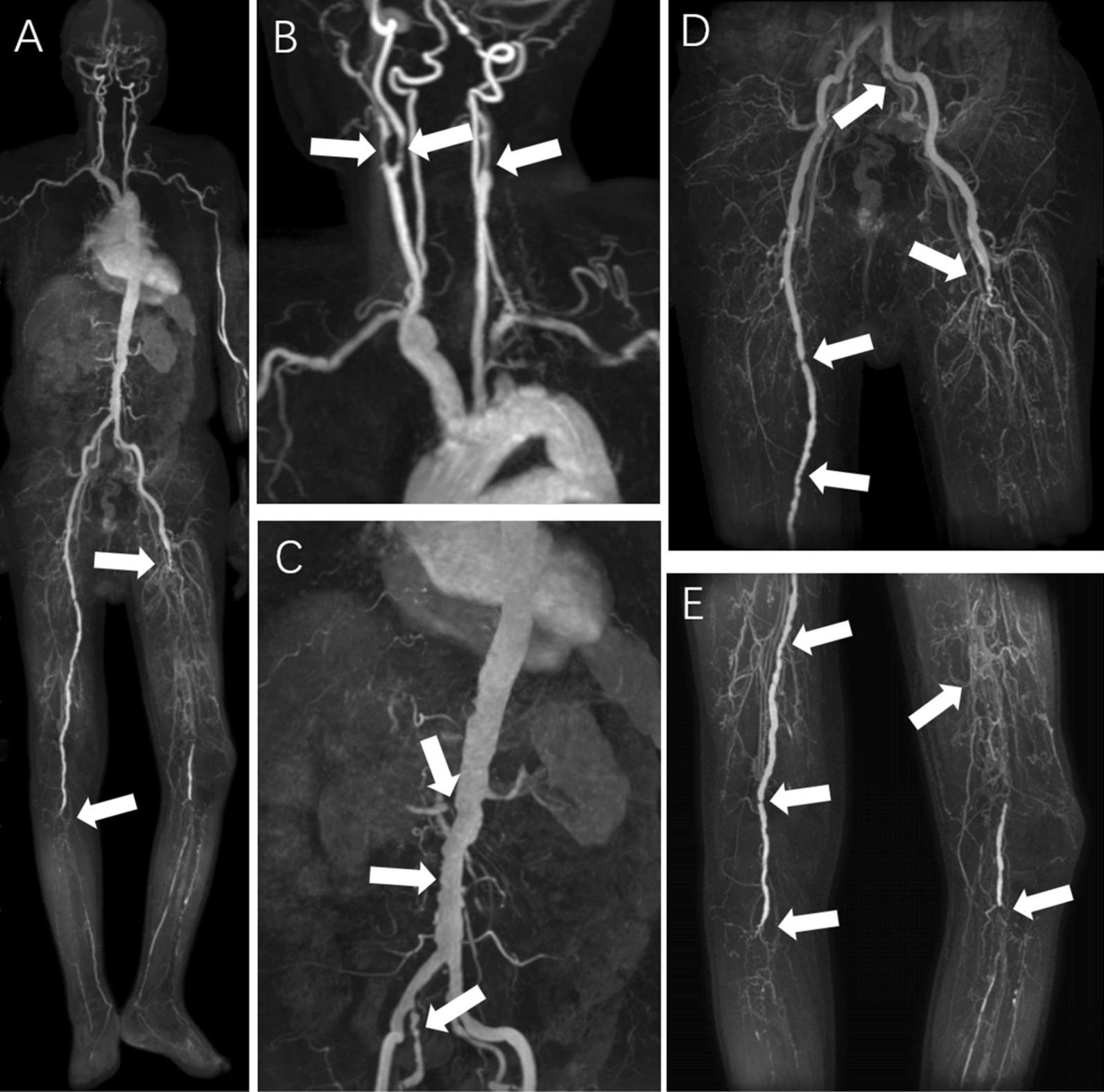
A male patient, 78 years old, had arteriosclerosis obliterans of the lower extremities, accompanied by gangrene. Multiple arterial segments were visualized with stenosis in whole-body subtractionless CS-WBMRA images (**A–E**, arrows). The left internal carotid artery, left femoral and popliteal arteries had complete occlusion; bilateral calf arteries had severe stenoses, accompanied by multiple collateral circulation with small vessels; right internal and external carotid arteries, and bilateral internal iliac arteries had moderate-to-severe stenoses

**Fig. 4:**
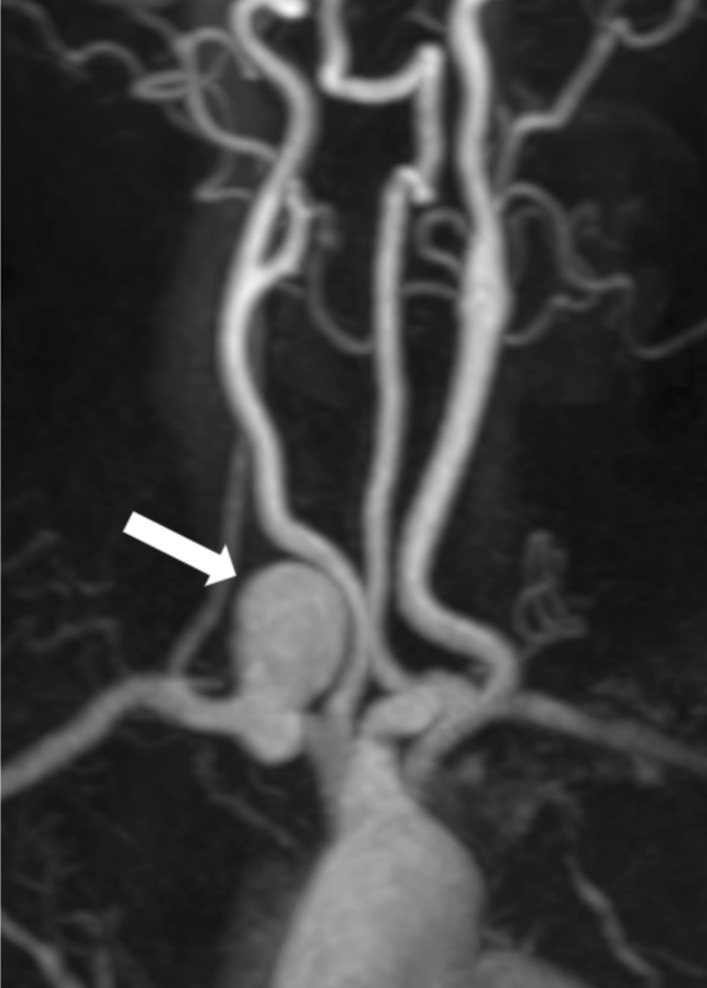
Station I of whole-body subtractionless CS-WBMRA MIP displaying an aneurysm in the right subclavian artery in a 50 years old female case

**Fig. 5:**
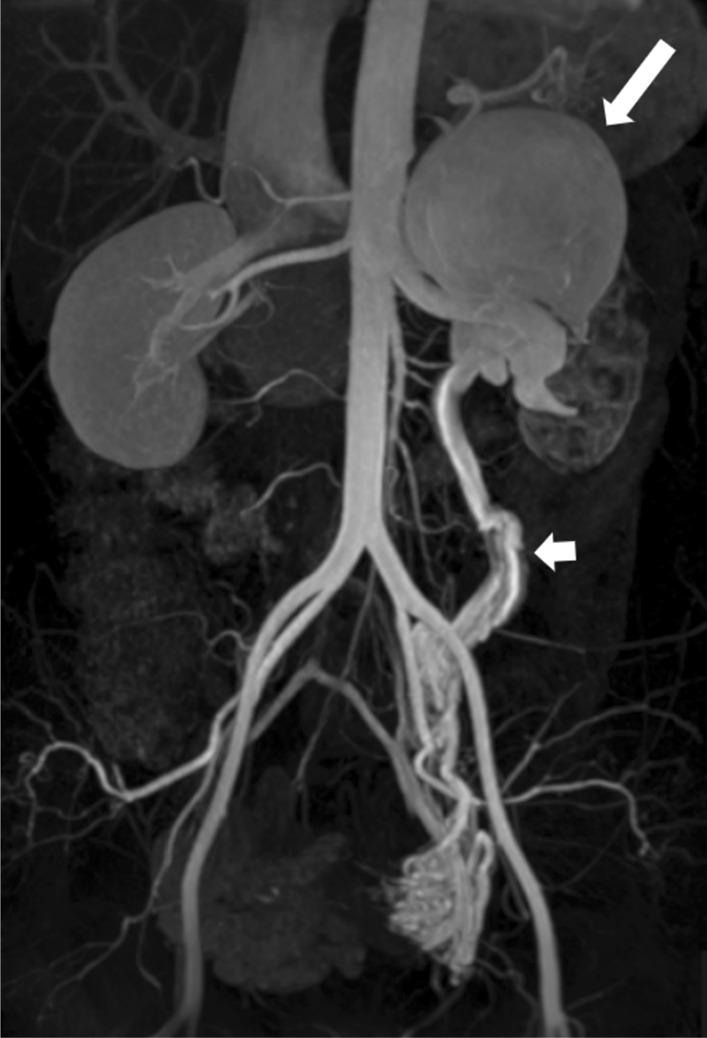
Abdominal region of whole-body subtractionless CS-WBMRA MIP showing arteriovenous malformation in the left renal region: the lumen of the left renal artery was enlarged, and the main trunk had an inner diameter of about 9 mm; the left renal vein was sac-shaped with contrast material evenly filled (long arrow), and its cross-sectional size was about 94 mm × 73 mm; the left gonadal vein was tortuous and thickened (short arrow), with an 8.4 mm inner diameter

All examinations were performed without complications, and no adverse reactions were reported. There were no statistically significant differences in patient weight and height between the CS-WBMRA and conventional-WBMRA protocols. Both MRA protocols yielded excellent results with acceptable diagnostic image quality in all the enrolled subjects (Fig. 2A, B). Of all patients, 75% (36/48) examined by CS-WBMRA showed systemic vascular diseases, including 34 patients with different stenosis grades in multiple arterial segments (Fig. 3), 1 had an aneurysm in the proximal right subclavian artery (Fig. 4), and 1 had an arteriovenous fistula in the left renal artery (Fig. 5).

In the CS-WBMRA protocol, 20/48 patients were imaged through 5-station coverage and 28/48 through 4-station coverage. In the conventional-WBMRA protocol, 14/38 patients were examined with 5-station coverage, and the remaining cases (24/38) were assessed with 4-station coverage.

### Quantitative image analysis

For overall analysis, apparent SNRs in the conventional-WBMRA protocol (214.98 ± 136.05) were higher (P < 0.001) than those of the CS-WBMRA protocol (164.90 ± 118.05), while the overall apparent CNRs were not statistically different between the two groups (CS vs conventional: 107.13 ± 72.323 vs 161.24 ± 118.64, P > 0.05). For segmental comparison, there were no significant differences (P > 0.05) in apparent SNRs and CNRs for common carotid arteries, common femoral arteries and popliteal arteries between the two groups (Table 4), except that the abdominal aorta had lower values in the CS-WBMRA group compared with the conventional-WBMRA group (P < 0.001).

**Table 4:** Apparent SNRs and CNRs for 4 anatomical regions in CS-WBMRA and conventional-WBMRA

Vessel segment	SNRcs	SNRc	P value	CNRcs	CNRc	P value
Common carotid arteries	111.52 ± 48.63	103.46 ± 96.83	0.192	106.4 ± 47.92	118.54 ± 68.57	0.358
Abdominal aorta	60.22 ± 27.73	147.56 ± 105.54	0.001	54.45 ± 19.22	125.54 ± 88.54	0.001
Common femoral arteries	252.65 ± 82.74	218.64 ± 157.64	0.234	255.6 ± 80.82	228.56 ± 96.54	0.170
Popliteal arteries	215.17 ± 76.4	220.54 ± 154.56	0.845	213.09 ± 68.67	248.54 ± 153.64	0.192
Overall comparison	164.90 ± 118.05	214.98 ± 136.05	< 0.001	161.24 ± 118.64	107.13 ± 72.323	0.073

*SNRcs* apparent signal to noise ratio of compressed sensing whole body MR angiography, *SNRc* apparent signal to noise ratio of conventional whole body MR angiography, *CNRcs* apparent contrast to noise ratio of compressed sensing whole body MR angiography, *CNRc* apparent contrast to noise ratio of conventional whole body MR angiography, *WBMRA* whole body MR angiography

### Qualitative image analysis

A total of 1776 and 1406 arterial segments in the CS-WBMRA and conventional-MRA protocols were available for the final qualitative evaluation. Overall scores for CS-WBMRA were significantly superior to those of conventional WMBRA (3.40 ± 0.60 vs 3.22 ± 0.55, Table 5). The subjective scores of 7 (out of 15) vessel segments had statistically significant differences (P < 0.05), with better image quality for CS-WBMRA compated to conventional-WBMRA (Table 5). The scores of the remaining 8 segments showed that both WBMRA methods yielded similar image quality levels (Table 3).

**Table 5:** Statistical analysis of the subjective image quality of arterial segments in conventional-WBMRA and CS-WBMRA

Arterial segment	Conventional-WBMRA	CS-WBMRA	P value
Right and left common carotid arteries	3.03 ± 0.16	3.3 ± 0.51	0.002
Right and left vertebral arteries	2.82 ± 0.46	3.13 ± 0.58	0.009
Brachiocephalic trunk	2.82 ± 0.39	3.09 ± 0.53	0.021
Thoracic aorta	3.66 ± 0.48	3.28 ± 0.59	0.004
Abdominal aorta	3.53 ± 0.6	3.41 ± 0.58	0.37
Right and left renal arteries	3.39 ± 0.72	3.24 ± 0.67	0.291
Superior mesenteric artery	3.34 ± 0.63	3.35 ± 0.53	0.99
Celiac artery	3.29 ± 0.57	3.41 ± 0.54	0.279
Right and left common iliac arteries	3.47 ± 0.6	3.39 ± 0.54	0.473
Right and left external iliac arteries	3.47 ± 0.6	3.52 ± 0.5	0.789
Right and left femoral arteries	3.08 ± 0.27	3.61 ± 0.49	0.000
Right and left popliteal arteries	3.13 ± 0.34	3.78 ± 0.41	0.000
Right and left anterior tibial arteries	3.29 ± 0.46	3.37 ± 0.68	0.268
Right and left posterior tibial arteries	3.11 ± 0.51	3.35 ± 0.64	0.032
Right and left peroneal arteries	3.11 ± 0.56	3.28 ± 0.66	0.123
Overall score comparison	3.22 ± 0.55	3.40 ± 0.60	< 0.001

*WBMRA* whole body MR angiography

**Fig. 6:**
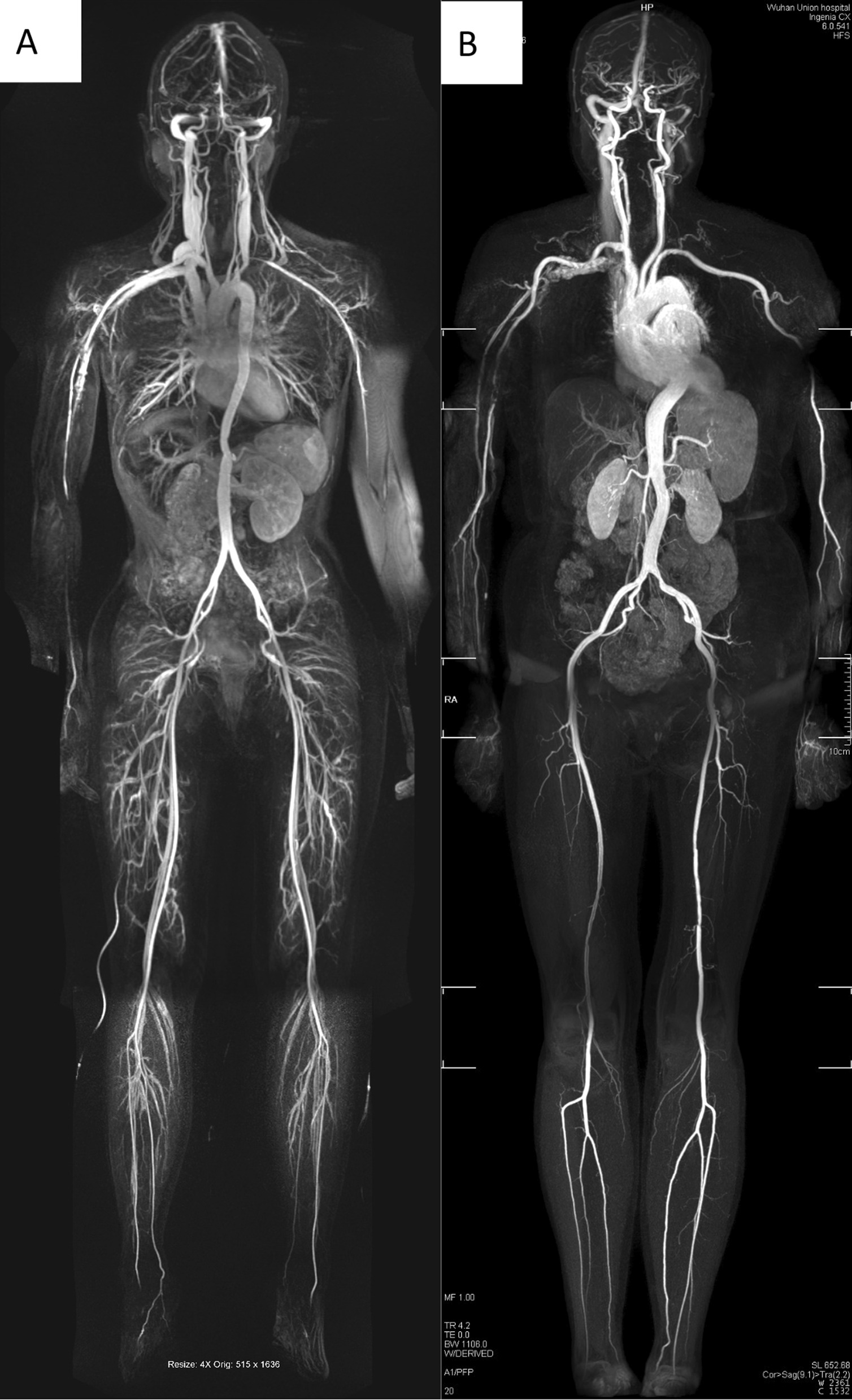
**A** Conventional-WBMRA; **B** cs-WBMRA; there were more vessel segments contaminated by veins in conventional-WBMRA due to longer scanning time, double contrast agent injection and misaligned subtraction because of patient motion. This is a great challenge for the accuracy of radiological diagnosis

For the evaluation of venous contamination in the CS-WBMRA protocol, 7 of 1776 (0.4%) vessel segments were contaminated severely by venous enhancement (score 3), including 1 in the renal artery, 1 in the tibioperoneal trunk, 3 in peroneal arteries and 2 in posterior tibial arteries); the remaining 99.6% (1769/1776) vessel segments showed minimal-to-moderate venous enhancement. On the other hand, severe venous contamination occurred in 317 of 1406 (22.5%) vessel segments with the conventional-WBMRA technique. There were more vessel segments contaminated by veins in the conventional-WBMRA group due to longer scanning time, double contrast injection, and misaligned subtraction because of motion, greatly decreasing the accuracy of radiological diagnosis (Fig. 6).

### Diagnostic accuracy

**Fig. 7:**
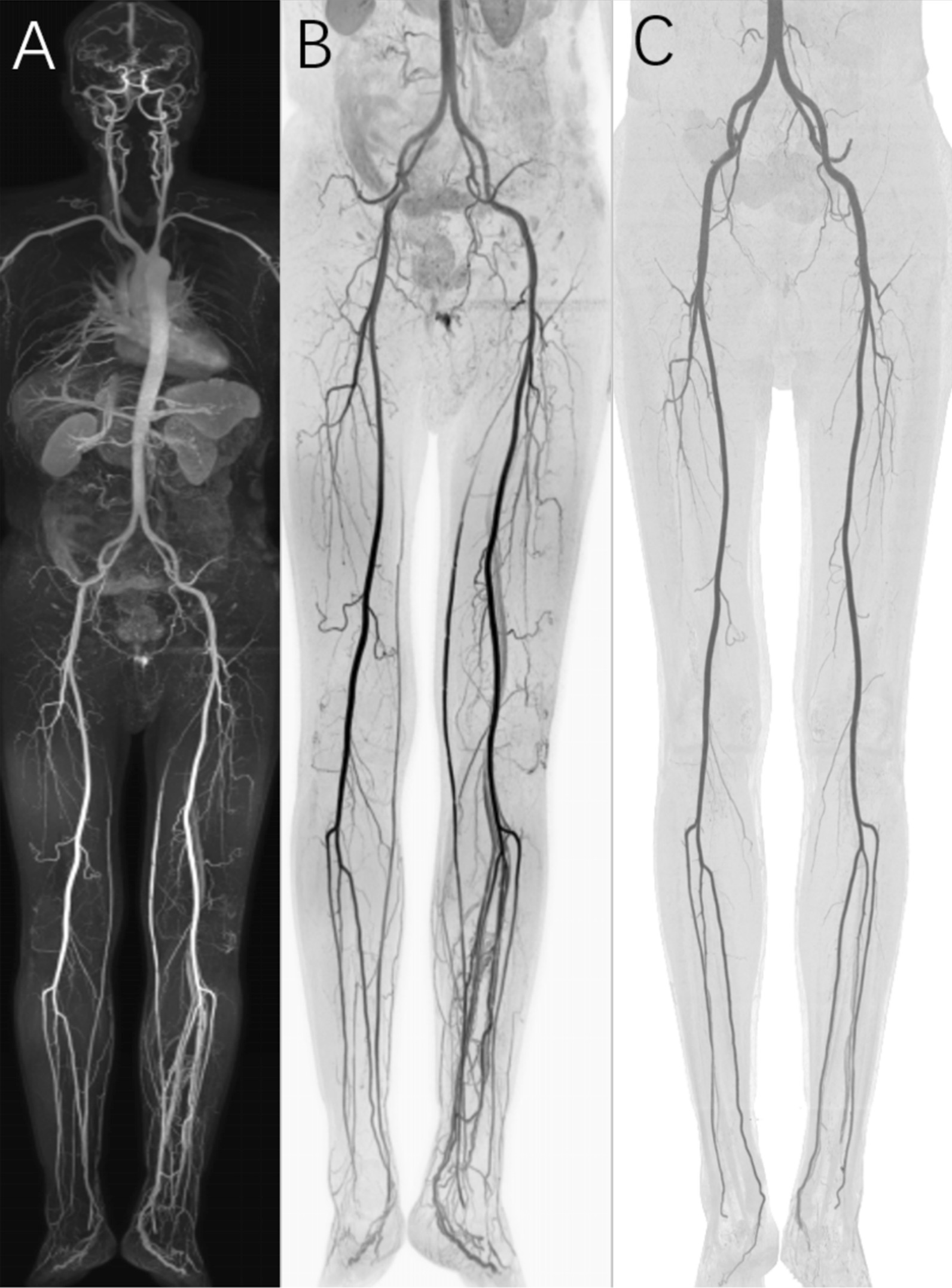
A male patient, 69 years old, presented with numbness in both lower limbs for a month. Whole-body subtractionless CS-WBMRA MIP (**A**) and inversed MIP of the lower extremity (**B**) showed normal arterial vascular tree of the whole body with early enhanced left calf vein. Inversed lower extremity CTA (**C**) confirmed the reliability of CS-WBMRA data

**Fig. 8:**
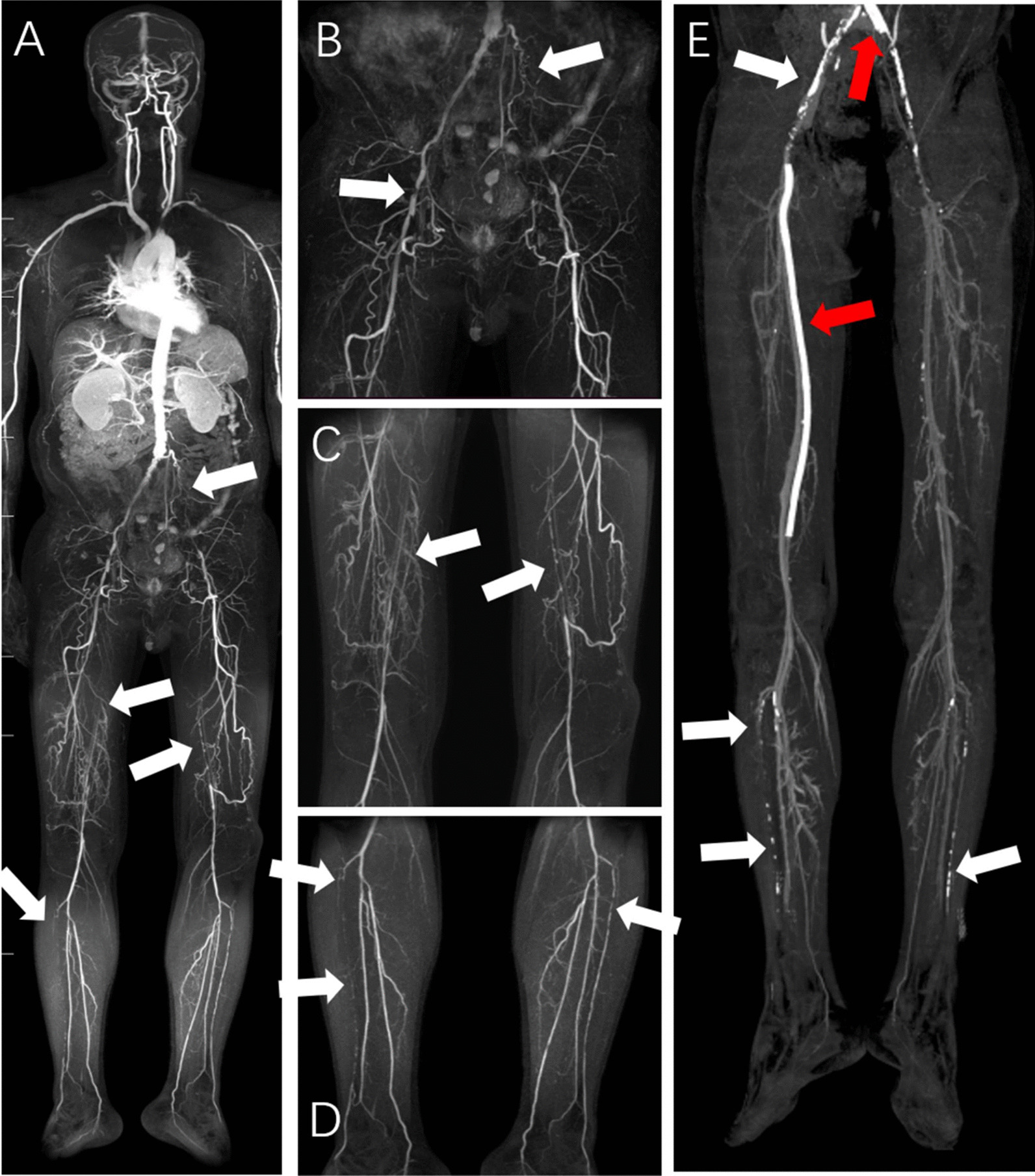
A male patient, 62 years old, had arteriosclerosis of lower extremities for several years. Whole-body subtractionless CS-WBMRA MIP (**A–D**) showed different grades of stenoses (white arrows). Three days after CS-WBMRA examination, the patient underwent stent placement surgery involving the left common iliac artery and the right superficial femoral artery. Corresponding CTA of the lower extremity (**E**) depicting the stents (red arrows) and CS-WBMRA findings clearly (white arrows)

**Fig. 9:**
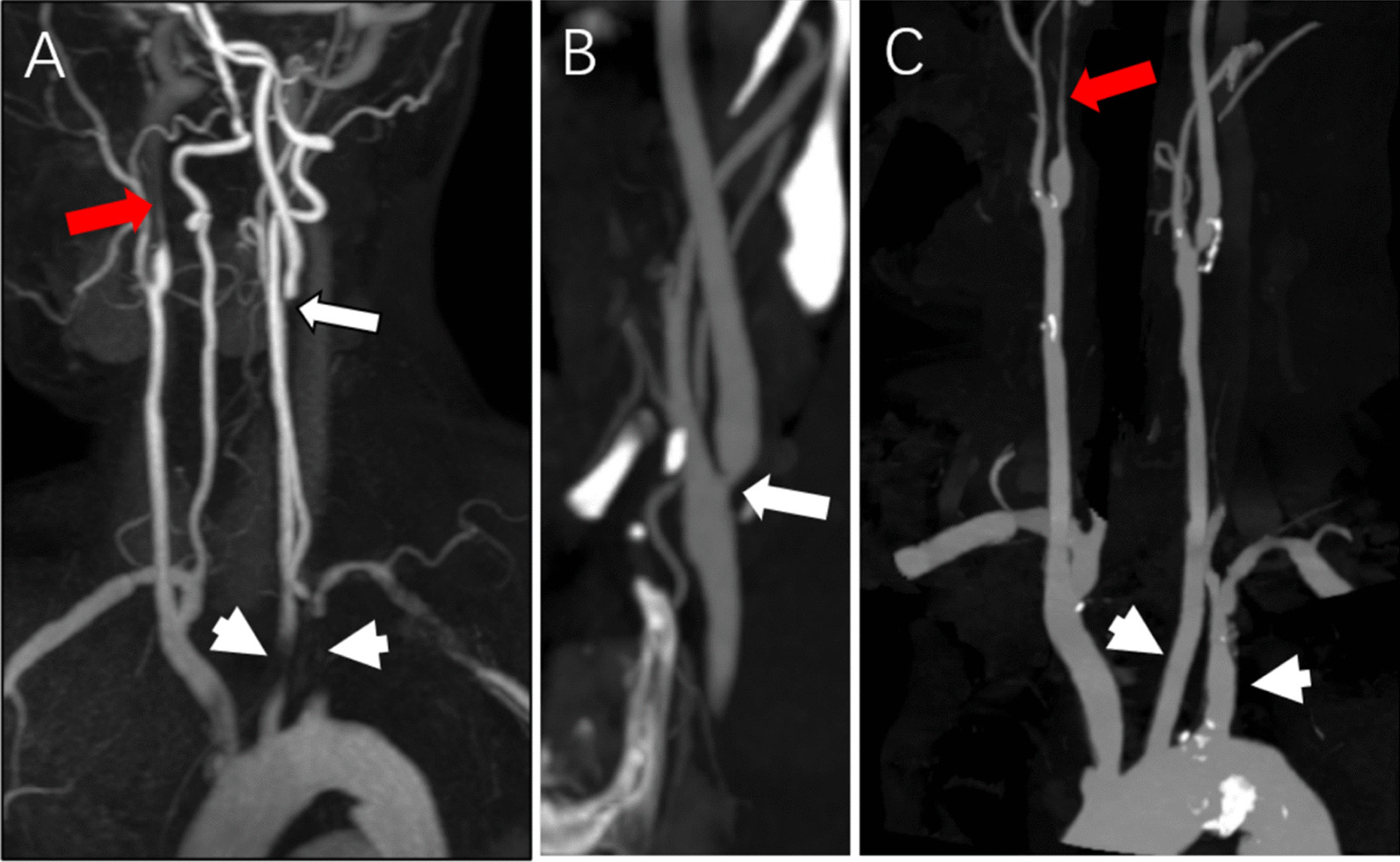
The same patient described in Fig. 6. In station I (head-neck region) of whole-body subtractionless CS-WBMRA MIP (**A**), an occlusion in the left internal carotid artery (long arrow) was detected but was graded as severely stenosed (> 90% stenosis) by carotid CTA (**B**, long arrow). In **A**, there seemed to be occlusions in the left common carotid artery and the left subclavian artery (short arrows), which were considered to be normal by CTA (short arrows in **C**). The distal part of the right internal carotid artery had severe stenosis (red arrow in **A**), causing a slight enlargement of the proximal part, which was confirmed by carotid CTA (red arrow in **C**)

Of the 17 patients who underwent CTA, 7 underwent lower extremity CTA, 2 underwent thorax-abdominal CTA, 1 underwent thorax CTA, 1 underwent abdominal CTA, 3 underwent renal CTA, 2 underwent head-neck CTA and 1 underwent pelvic CTA, resulting in a total of 244 arterial segments that were assessed by both CS-WBMRA and CTA. The abnormalities detected by CTA are shown in Table 6. For a comparative analysis, 93.3% (227/244) of segments assessed by CS-WBMRA had the same rating scores as those examined by CTA (Figs. 7, 8). Of the remaining 17 segments with different scores between CS-WBMRA and CTA, only 3 were overestimated: 1 in the left common carotid artery with normal in CTA (score 1) was considered an arterial occlusion (score 4) on CS-WBMRA scans, caused by susceptibility artifacts (Fig. 9). Two segments (1 in the proximal left subclavian artery and 1 in the IMA) were overestimated as severely stenosed (score 3) but normal on CTA scans (score 1). Six segments assessed as occlusion by CS-WBMRA were shown to be severe stenoses in CTA, four segments graded as normal by CTA were assessed as mild-to-moderate stenoses by CS-WBMRA, and four segments were over-graded as severe stenoses by CS-WBMRA, but these segments were assessed as mild-to-moderate stenoses by CTA. Thus, the overall sensitivity and specificity of CS-WBMRA in the detection of significant arterial stenoses (luminal narrowing > 50%) were 100.0% and 96.7%, respectively.

**Table 6:** Assessment of peripheral vascular diseases in 17 patients by CTA and WBMRA on a segment-basis

Arterial segment	Stenosis < 50%		Stenosis 50–99%		Occlusion		Aneurysm or arteriovenous malformation	
	CTA	MRA	CTA	MRA	CTA	MRA	CTA	MRA
Internal carotid artery	5	5	0	0	1	1	0	0
External carotid artery	6	6	0	0	0	0	0	0
Vertebral artery	5	5	1	1	0	0	0	0
Common carotid artery	6	4	0	1	0	1	0	0
Brachiocephalic trunk	3	3	0	0	0	0	1	1
Thoracic aorta	3	3	0	0	0	0	0	0
Suprarenal abdominal aorta	5	5	0	0	0	0	0	0
Infrarenal abdominal aorta	5	4	0	1	0	0	0	0
Renal artery	9	9	1	0	0	1	1	1
Superior mesenteric artery	5	4	0	1	0	0	0	0
Inferior mesenteric artery	5	4	0	1	0	0	0	0
Celiac artery	5	5	0	0	0	0	0	0
Common iliac artery	25	25	0	0	1	1	0	0
External iliac artery	17	17	1	0	1	2	0	0
Common femoral artery	16	16	2	2	0	0	0	0
Proximal superficial femoral artery	16	16	2	1	2	3	0	0
Distal superficial femoral artery	14	14	0	0	1	1	0	0
Popliteal artery	13	13	1	1	2	2	0	0
Tibioperoneal trunk	13	11	0	2	2	2	0	0
Anterior tibial artery	10	10	1	0	4	5	0	0
Peroneal artery	12	12	2	1	4	5	0	0
Posterior tibial artery	11	11	1	0	3	4	0	0
Total	209	202	12	12	21	28	2	2

*WBMRA* whole body MR angiography

### Time and contrast dose

The total cumulative measurement time of CS-WBMRA arterial sequences was 60.4 s, vs 193.8 s for conventional-WBMRA sequences. Considering the time for the preparation of patient localization, automatic table movement, B0 shimming, and instructions for patients to hold their breath, the average total CS-WBMRA examination time from patient localization to scanning end was 7 min. The corresponding time for the conventional-WBMRA protocol was 20 min. The total contrast agent volume was significantly reduced in CS-WBMRA compared with the conventional-WBMRA protocol (18.7 ± 3.5 ml, 37.2 ± 5.4 ml, P = 0.008). However, CS-WBMRA required an additional 10 min for image reconstruction from the end of image acquisition, while conventional-WBMRA could complete image reconstruction immediately (< 30 s) after the scanning.

## Discussion

In the current study, we demonstrated the feasibility and clinical utility of a subtractionless CS-WBMRA protocol that requires only a single injection of contrast material with only 0.15 mmol/kg contrast agent for the visualization of whole-body arterial vasculature, with comparable image quality to conventional-WBMRA. Comparative analysis between CS-WBMRA and available CTA supported a high diagnostic performance for CS-WBMRA protocol for arterial diseases.

The feasibility of the CS-WBMRA protocol described in this study could be attributed to a combination of advances in multiple techniques, including high-element receiver coils, automatic table movement, automatic coil selection techniques, multi-echo Dixon water-fat separation and the CS-based imaging acceleration technique, which facilitate the rapid acquisition of each station by WBMRA in pace with the arrival of the contrast bolus. The fast flow of the contrast agent between stations I and II (head-neck region to thorax area) leaves a very short pure arterial time window between these two regions, typically below 5 s. In the previously applied approach, when the contrast agent is injected as a single bolus, venous contamination to the later acquired station between I and II would be severe and thus impair the corresponding image quality, leading to failed acquisition of the arterial MRA. In the novel protocol, the scan time of stations I and II were limited to only 9.8 s and 11.7 s, respectively, by applying a CS acceleration factor of 10, which was used together with a reverse-centric k-space technique to match the pace of the fast-moving contrast material in vessels, allowing for accurate depiction of arterial bolus arrival and efficient use of peak contrast enhancement in arterial vessels.

In a previous whole-body MRA study with a single injection of the contrast agent [28], 4 stations were used for complete coverage, and acquisition times for stations I and II were set to be 12 s and 15 s, respectively, by applying parallel imaging in both phase- and section-encoding directions (iPAT). While the imaging time per station (I and II) detected in this study was approximately 17% shorter than described previously, the contrast agent dose was considerably reduced by approximately 40%, which would potentially reduce risks associated with gadolinium-based MR contrast agents, including nephrogenic systemic fibrosis [29] and gadolinium-related deposition in the central nervous system [30]. This reduction could also benefit patients by decreasing the cost of contrast agents with reduced total volume required.

Moreover, the multi-echo two-point Dixon technique (mDixon) is known to improve vasculature conspicuity by providing fat-suppression without the need for subtraction between post- and pre-contrast images, in turn avoiding the possibility of misregistration artefacts resulting from patient respiratory movement/motion [12]. Compared with the subtracted method, the mDixon technique achieves high apparent SNR and spatial resolution as well as vessel-to-background contrast [12].

Although SNR would decrease to some extent by the high acceleration of CS-SENSE, our data showed no significant differences in the apparent SNRs and CNRs of common carotid arteries, common femoral arteries, and popliteal arteries between the two groups. However, the apparent SNR and CNR of the abdominal aorta were reduced in the CS-WBMRA group compared with the conventional-WBMRA method. This might be because the first peak value of contrast in station II occurred a few seconds after station I was scanned in the CS-WBMRA group. In the conventional-WBMRA group, the peak value of contrast was reached at the acquisition of the central K-space of station II, resulting in improved arterial enhancement. However, the subjective image quality for 99.6% of the examined vessel segments was satisfactory for diagnosis, supporting the feasibility of this method in clinic.

With the optimization of scanning parameters and proper contrast injection strategy, venous contamination was almost minimal-to-moderate in 1769 out of the 1776 vessel segments examined, implying that this method would not cause substantial impairment in image quality, corroborating another ultrafast first-pass whole-body MRA study of the PI technique[28]. Only 7 vessel segments were contaminated severely by venous enhancement, including 6 (85.7%, 6/7) that were localized in the lower leg. Because vessel diameters in the lower leg were relatively smaller, we employed a higher spatial resolution of station V (0.9 × 0.9 × 1.0 mm^3^) compared with those of other stations to evaluate the vessel segments accurately and to decrease the interference of enhanced veins to arterial lesions. As for other indexes, the CS factor of the lower leg was 8, and the scan time was 15.6 s, i.e., a little longer than the time used for the upper leg. This modified approach aimed to make arteries and related branches in the lower leg station fill with adequate contrast agent and to guarantee a sufficient SNR for arteries.

In a recent study, Weiss et al. [12] compared the feasibility and robustness of subtractionless single-pass peripheral MRA and the subtracted method in terms of SNR and vessel-to-background contrast, but the sample size was relatively small (10 patients) and whole-body coverage and diagnostic accuracy were not involved. A previous study [28] focused on image quality evaluation of ultrafast whole-body MRA with parallel imaging at 3 T, including 23 patients; the evaluation was performed based on subjective scores and objective analyses, but diagnostic accuracy was not investigated. The current study enrolled a larger number of patients for a more comprehensive evaluation of the technique, with CTA considered the gold standard for 17 patients in the cohort. The data demonstrated a high accuracy for CS-WBMRA in the detection of significant arterial stenosis (a sensitivity of 100.0% and a specificity of 96.7%), which was higher than previously reported (sensitivities and specificities of 92–95% and 88–97%, respectively) [8, 31].

### Limitations

There were some limitations in this study. First, only 17 cases in the CS-WBMRA group underwent partial CTA examination, which might reduce the statistical validity of this study. Further cases examined by CTA/DSA and CS-WBMRA simultaneously should be assessed in the future for comparing diagnostic ability to detect arterial diseases between the two methods. Secondly, CS-WBMRA required an additional 10 min for reconstructing arterial and venous images of whole-body coverage, which limits patient throughput in the clinical setting, although no failure of image scanning or reconstruction occurred in the current study. This may be addressed with a higher-performance computer or improved reconstruction algorithms. Thirdly, bilateral arteries of the arms were not included in the present study. Fourthly, arterial assessment was not performed for intracranial vessels and coronary arteries for the following reasons: (1) the spatial resolution of station I (1.1 × 1.2 × 1.2 mm^3^) was not enough to accurately depict stenotic diseases in small intracranial vessels; (2) it remains challenging to visualize coronary arteries by the MRA method [28]. Fifthly, the objective evaluation metrics SNR and CNR might be affected by acceleration techniques (SENSE and CS-SENSE).

## Conclusion

In this study, we demonstrated that a one-stop subtractionless CS-accelerated WBMRA protocol could provide higher image quality and diagnostic performance for whole-body arterial vasculature compared with the conventional subtracted-MRA method and available CTA, which could reduce the complexity of whole-body CE-MRA examination and allow a faster workflow for visualizing and depicting the whole-body arterial vasculature in just 10 min for clinical use.

## Data Availability

The datasets and analysis tools used during this study are available from the corresponding author upon reasonable request.

## Abbreviations

WBMRA: Whole body magnetic resonance angiography
CS: Compressed sensing
mDixon: Multi-echo Dixon
CE-MRA: Contrast-enhanced magnetic resonance angiography
CTA: Computed tomography angiography
PI: Parallel imaging
SENSE: Sensitivity encoding
